# Delmarva (DMV/1639) Infectious Bronchitis Virus (IBV) Variants Isolated in Eastern Canada Show Evidence of Recombination

**DOI:** 10.3390/v11111054

**Published:** 2019-11-13

**Authors:** Mohamed S. H. Hassan, Davor Ojkic, Carla S. Coffin, Susan C. Cork, Frank van der Meer, Mohamed Faizal Abdul-Careem

**Affiliations:** 1 Faculty of Veterinary Medicine, University of Calgary, Health Research Innovation Center 2C53, 3330 Hospital Drive NW, Calgary, AB T2N 4N1, Canada; msh.hassan@ucalgary.ca (M.S.H.H.); sccork@ucalgary.ca (S.C.C.); fjvander@ucalgary.ca (F.v.d.M.); 2 Animal Health Laboratory, University of Guelph, Guelph, ON N1G 2W1, Canada; dojkic@uoguelph.ca; 3 Cumming School of Medicine, University of Calgary, Health Research Innovation Center 2C53, 3330 Hospital Drive NW, Calgary, AB T2N 4N1, Canada; cscoffin@ucalgary.ca

**Keywords:** Infectious bronchitis virus (IBV), recombination, complete genome sequence, phylogenetic analysis, chicken, Canada

## Abstract

Infectious bronchitis virus (IBV) infection in chickens can lead to an economically important disease, namely, infectious bronchitis (IB). New IBV variants are continuously emerging, which complicates vaccination-based IB control. In this study, five IBVs were isolated from clinical samples submitted to a diagnostic laboratory in Ontario, Canada, and subjected to detailed molecular characterization. Analysis of the spike (S)1 gene showed that these five IBVs were highly related to the Delmarva (DMV/1639) strain (~97.0% nucleotide sequence similarity) that was firstly isolated from an IB outbreak in the Delmarva peninsula, United States of America (USA), in 2011. However, the complete genomic sequence analysis showed a 93.5–93.7% similarity with the Connecticut (Conn) vaccine strain, suggesting that Conn-like viruses contributed to the evolution of the five Canadian IBV/DMV isolates. A SimPlot analysis of the complete genomic sequence showed evidence of recombination for at least three different IBV strains, including a Conn vaccine-like strain, a 4/91 vaccine-like strain, and one strain that is yet-unidentified. The unidentified strain may have contributed the genomic regions of the S, 3, and membrane (M) genes of the five Canadian IBV/DMV isolates. The study outcomes add to the existing knowledge about involvement of recombination in IBV evolution.

## 1. Introduction

Infectious bronchitis (IB), an acute and highly contagious disease, is characterized by respiratory, renal, or reproductive diseases in chickens of all ages [1]. Here, the infectious bronchitis virus (IBV) is the causative agent and is an enveloped, positive-sense single-stranded ribonucleic acid (RNA) virus which belongs to the genus *Gammacoronavirus* of the family *Coronaviridae* [2]. The size of the IBV genome is approximately 27.6 kilo-bases (kb) and the genome encodes at least 10 open reading frames (ORFs), flanked by two untranslated regions (UTR), and is organized as follows: 5′ (UTR)-1a-1ab-S-3a-3b-envelope (E)-M-5a-5b-nucleocapsid (N)-3′ UTR [3]. Ribosomal frameshifting from the 1a reading frame into the 1ab ORF at the 5′ region generates two polyproteins (1a and 1ab) which are then cleaved by two types of virus-encoded proteases. The process generates 15 non-structural proteins (nsps), nsps 2-16, that are necessary for replication and pathogenicity [3,4]. The remaining 3′ end of the genome encodes structural components, including S, M, E, and N proteins, as well as small non-structural accessory proteins (3a, 3b, 4b, 4c, 5a, 5b and 6b) with unclear functions [5].

The S protein undergoes post-translational cleavage, resulting in the S1 and S2 subunits. The glycoprotein S1 carries the receptor binding site and plays a major role in tissue tropism and the induction of protective immunity [6]. Consequently, S1 gene sequence analysis is used by many laboratories for IBV genotyping [7]. The high sequence diversity of the IBV S1 subunit results in the emergence of new virus genotypes, serotypes, and variants with variable geographic distributions [8].

In addition to accumulation of mutations, recombination events are also common during IBV replication, which result in the emergence of major IBV variants. It is possible that the recombination events occur at multiple sites of the IBV genome, leading to rapid viral evolution [9,10,11,12]. For example, a recombination event that involved the 5′ terminal end of the S1 gene containing hypervariable regions I and II has driven an IBV isolate of 4/91 genotype to be more serologically related with H120 strain [13]. Further, it has been suggested that the acquisition of the 3′ end of the N gene and the 3′ UTR from a strain of different genotype, through recombination, may alter the replication efficiency of an IBV isolate [14].

Despite the routine vaccination of Canadian poultry flocks against IB, repeated outbreaks occur [15,16]. Viruses similar or related to variant IBVs described in the USA are continuously reported in Canada. Characterization of the partial S1 gene of strains IBV-ON2, IBV-ON3, and IBV-ON5 demonstrated their high sequence identity with strain CU-82792, and that IBV-ON4 shared a 98.7% nucleic acid identity with strain PA/1220/9 [17]. Later, US variant-like virus strains (California 1734/04, California 99, CU_82792, Pennsylvania 1220/98, and Pennsylvania Wolg/98) were identified in a study aimed at genotyping IBV isolates in Canada that were circulating between 2000 and 2013 [18]. Based on partial or full S1 gene sequences, IBV variants such as CA/1737, DE/072, PA/Wolgemuth/98, DMV/1639 and Massachusetts (Mass) were detected in clinical samples [15,19].

Although the classification of IBV variants based on partial or full S1 gene sequences is feasible, in depth molecular analysis of IBV variants requires the examination of the complete genome of the IBV. Previously, two Mass IBV variants isolated from western Canada were characterized using whole genome sequencing [20]. In the current study, the genomic makeup of DMV/1639-like isolates from diagnostic samples are characterized in detail, and several potential recombination sites are identified.

## 2. Materials and Methods

### 2.1. Virus Isolation

Specific pathogen free (SPF) fertile chicken eggs were used for IBV isolation. The use of embryonated eggs for virus isolation has been approved by the Health Science Animal Care Committee (HSACC) of the University of Calgary, Calgary, Canada. Five diagnostic samples, which were characterized by partial S1 gene sequencing to be related to DMV/1639 strain, were received from the Animal Health Laboratory (AHL), University of Guelph, Canada, for virus isolation. The samples were collected from different farms located in Ontario, Canada, and the relevant history for each sample is summarized in Table 1. Virus isolation was carried out by inoculation of 9–11-day-old embryonated SPF eggs with 150 µL of 10% tissue homogenates or virus transport medium. After 48 h of incubation at 37.6 °C, the eggs were refrigerated 12 h, then the allantoic fluid was collected and stored at –80 °C. Allantoic fluid was serially passaged in the embryonated SPF eggs 2–4 times in order to obtain high virus titers.

### 2.2. RNA Extraction and Complementary Deoxyribonucleic Acid (cDNA) Synthesis

Total RNA was extracted from 250 µL of the harvested allantoic fluid using the Trizol LS^®^ reagent (Ambion, Invitrogen Canada Inc., Burlington, ON, Canada), according to the manufacturer’s protocol. A Nanodrop 1000 spectrophotometer (Thermo Scientific, Wilmington, DE, USA) at a 260 nm wavelength was used to determine the concentration of the extracted RNA, and the quality was determined using the Nanodrop spectrophotometer, based on the 260/280 absorbance ratio. Approximately 1000 ng of extracted RNA was used for cDNA synthesis using random primers (High Capacity Reverse Transcription Kit™, Applied Biosystems, Invitrogen Canada Inc., Burlington, ON, Canada) according to the manufacturer’s instructions.

### 2.3. Real-Time PCR and Sequencing

In order to confirm the propagation of the virus in allantoic fluid, a previously described real-time PCR assay [21] was used to amplify a conserved sequence of 206 bp within the IBV-N gene using two primers, namely, Fw-5′GACGGAGGACCTGATGGTAA-3′ and Re-5′CCCTTCTTCTGCTGATCCTG-3′. Embryo passages with high IBV RNA concentrations (Cq value <20) were sent for whole genome sequencing at the Faculty of Veterinary Medicine, University of Montreal, Montreal QC, Canada, using a Miseq platform (Illumina corp, San Diego, CA, USA).

### 2.4. Genotyping and Comparison of S1 Gene Sequences

The complete sequences of the S1 gene of our five IBV isolates were phylogenetically analyzed against 86 reference sequences (Table S1), including vaccine strains, other Canadian field IBV strains [15,17,18,22,23], and representative sequences for each genotype and lineage as recommended [7] (available online, https://www.ncbi.nlm.nih.gov/genbank/). The complete S1 gene sequences were aligned using Clustal Omega [24], and the phylogenetic tree was inferred using the maximum likelihood method available in RAxML [25], with 1000 bootstrap replicates for branch support. The multiple sequence alignment and the phylogenetic tree construction were implemented using Geneious^®^ v10.2.6 (https://www.geneious.com/).

### 2.5. Characteristics of the Complete Genome

The ORF predictions of our five IBV isolates were carried out using the ORF-finder program (https://www.ncbi.nlm.nih.gov/orffinder/). The complete genome sequences of our five IBV isolates were submitted to Basic Local Alignment Search Tool (BLAST) in the National Center for Biotechnology Information (NCBI) database (https://blast.ncbi.nlm.nih.gov/Blast.cgi) to identify the IBV strains with the highest sequence identities. For further analysis, 364 complete genome sequences were downloaded from GenBank (https://www.ncbi.nlm.nih.gov/genbank/), and CD-HIT-EST [26] was used to cluster sequences with over a 97% identity match. After clustering, 146 representative sequences and the sequences from our five IBV isolates were aligned using multiple alignment with fast Fourier transformation (MAFFT) [27], and phylogenetic analysis was carried out using the maximum likelihood method in RAxML using Geneious^®^ v10.2.6.

The complete genome sequences from our five IBV isolates were aligned with the Conn vaccine strain (KF696629) using MAFFT. Sequence segments that were different from those of the Conn vaccine strain were submitted to BLAST to identify the IBV sequences with the highest percentages of identity. On the basis of the BLAST search results, the complete genomic sequences of our five IBV isolates were again aligned with those of the Conn vaccine strain and 4/91 vaccine strain (KF377577) using MAFFT.

Similar analysis included isolate GA9977/2019 (MK878536), which is a DMV/1639 IBV strain that was isolated from a broiler chicken farm in Georgia, USA, in 2019 [28]. The complete GA9977/2019 genome was compared to the sequences of our five IBV isolates and reference strains for homology analysis. The complete genomic sequence of isolate GA9977/2019 was aligned with those of the Conn vaccine strain and the 4/91 vaccine strain using MAFFT.

### 2.6. Recombination Analysis

To identify the presence of recombination events, a multiple sequence alignment was performed using the complete genome sequences of our five IBV isolates and the Conn and 4/91 vaccine strains. This sequence alignment was introduced into Simplot [29]. The nucleotide identity was calculated by the Kimura 2 parameter substitution model, with a window size and step size of 1000 and 50 nucleotides, respectively. Furthermore, pairwise comparison of the complete genome sequences of our five IBV isolates was performed with the Conn vaccine, 4/91 vaccine, and H120 (FJ888351) strains to confirm the recombination breakpoints. The phylogenetic trees of the putative recombinant fragments were constructed using PhyML [30] in Geneious^®^ v10.2.6.

### 2.7. GenBank Accession Numbers

The complete genomic sequences of our five IBV isolates, namely, IBV/Ck/Can/17-035614, IBV/Ck/Can/17-036989, IBV/Ck/Can/18-048192T, IBV/Ck/Can/18-048430, and IBV/Ck/Can/18-049707 were deposited into GenBank (accession numbers MN512434, MN512435, MN512436, MN512437 and MN512438, respectively).

## 3. Results

### 3.1. Phylogenetic Analysis and Sequence Comparison Based on the S1 Gene

**Figure 1:**
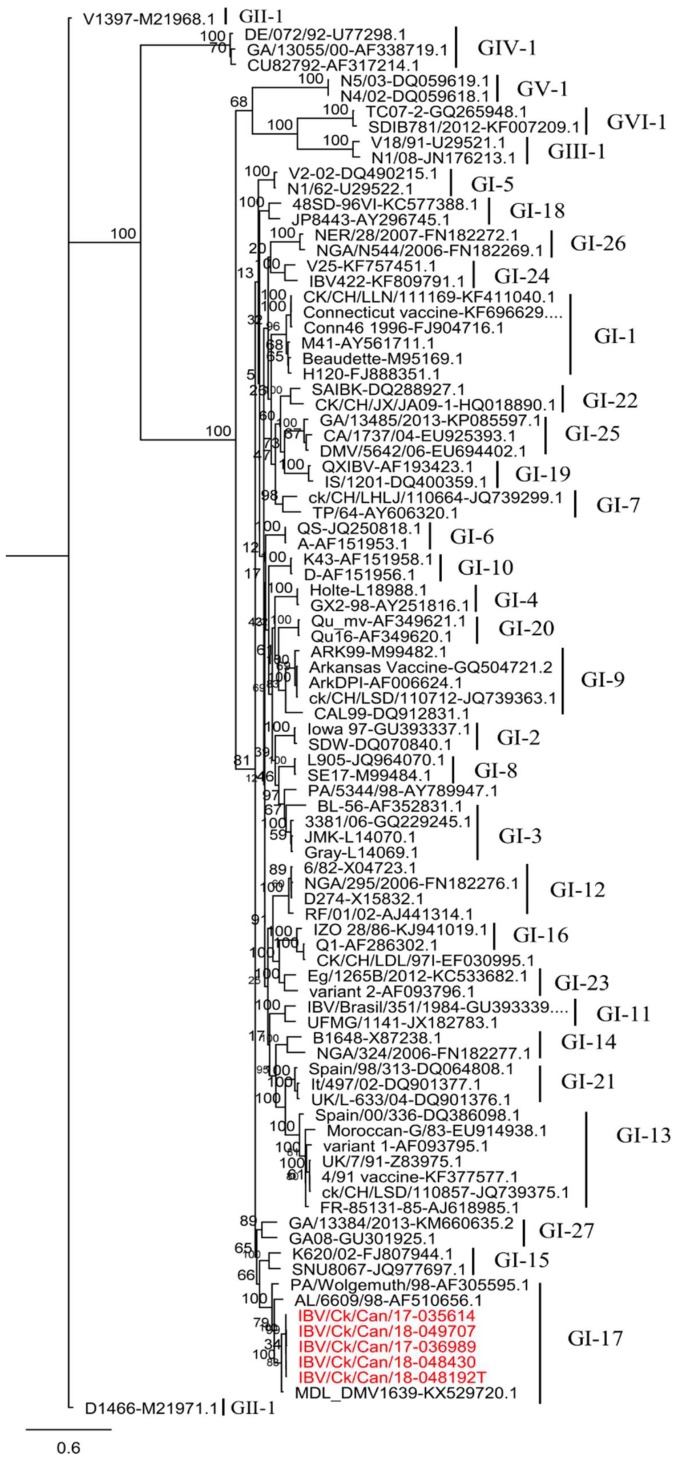
Randomized accelerated maximum likelihood (RAxML) phylogenetic tree based on the complete nucleotide sequences of S1 genes of our five IBV isolates and 86 reference sequences. All genotypes of representative IBV strains are indicated on the right side. Our five IBV isolates are marked in red color.

The S1 gene sequences of our five IBV isolates were clustered within the lineage GI-17 (Figure 1). This lineage consists of IBV viruses isolated in the USA from respiratory, nephropathogenic, and reproductive diseases outbreaks [7]. 

The S1 gene sequences from our five IBV isolates showed a high degree of nucleotide identity (99.4–99.9%). They were 96.4–96.7% identical to the MDL_DMV1639 (KX529720) strain, and, conversely, they were distinctly separate from the Mass and Conn strains, with only 77.2–77.3% nucleotide similarity in this case. Nucleotide and amino acid pairwise comparisons between the S1 genes of our five IBV isolates and other strains affecting poultry industry in Canada are listed in Table 2.

### 3.2. Complete Genome Sequences

**Figure 2:**
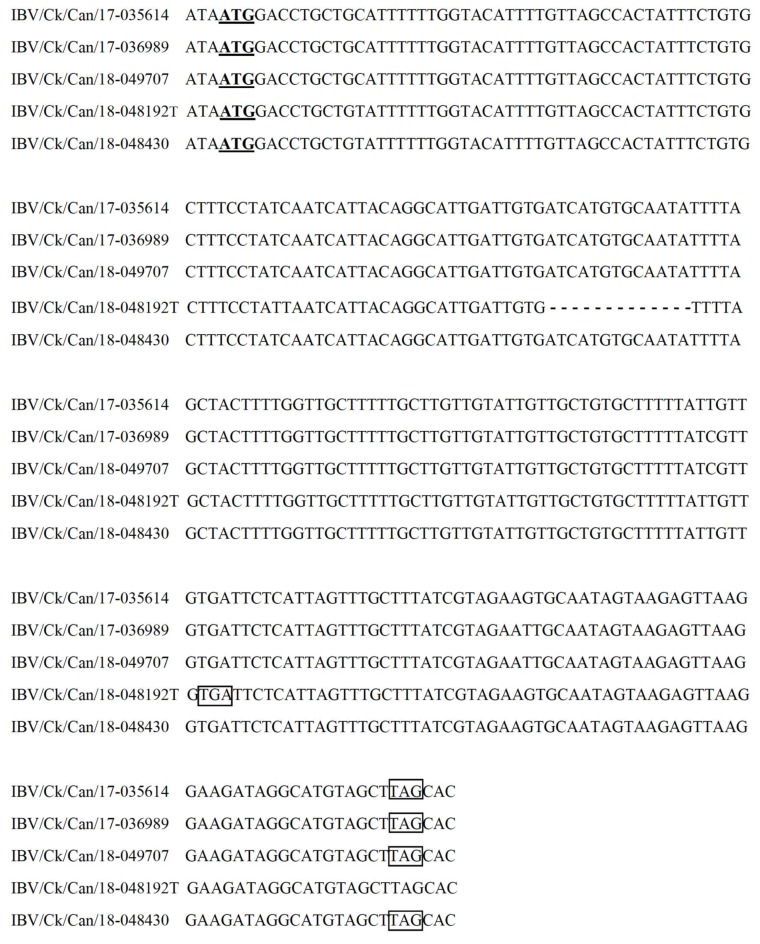
Sequence alignment of open reading frame (ORF) 6b from our five IBV isolates. The deletion in gene 6b of isolate IBV/Ck/Can/18-048192T results in a truncated 6b protein. Sequences of isolates IBV/Ck/Can/17-035614, IBV/Ck/Can/17-036989, IBV/Ck/Can/18-048430, and IBV/Ck/Can/18-049707, as representatives of the normal 6b gene, were compared. The ATGs with a single underline in boldface are the start codons of 6b gene. TGA and TAGs in boxes are the termination codons of 6b gene in IBV/Ck/Can/18-048192T and the other four isolates, respectively. A 13-nucleotide sequence was deleted in isolate IBV/Ck/Can/18-048192T (represented as –).

The complete genome sequences of IBV isolates IBV/Ck/Can/17-035614, IBV/Ck/Can/17-036989, IBV/Ck/Can/18-048192T, IBV/Ck/Can/18-048430, and IBV/Ck/Can/18-049707 were 27,710, 27,708, 27,638, 27,684, and 27,708 nucleotides in length, respectively. Thirteen ORFs (5′ -1a-1ab-S-3a-3b-E-M-4b-4c-5a-5b-N-6b-3′) were predicted. A deletion of thirteen nucleotides was detected in the reading frame of the 6b gene of isolate IBV/Ck/Can/18-048192T, compared to the other four IBV isolates (Figure 2). The deletion of 13 nucleotides led to a frameshift, and, therefore, if the 6b ORF is expressed, it results in a truncated protein with 47 deduced amino acids. The ORFs and genomic regions present in our five IBV isolates are listed in Table 3. The gene locations for the nsps in ORF 1a and 1ab are shown in (Table S2).

**Figure 3:**
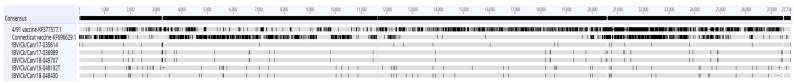
Alignment of the complete genome sequences of our five IBV isolates, the Connecticut vaccine, and 4/91 vaccine strains, which was performed using MAFFT. Nucleotide sequence disagreements at the indicated positions are shown in black, while nucleotide sequence agreements are shown in gray.

Our five IBV isolates were nearly identical among themselves, with an overall nucleotide sequence identity of 99.4–100%. A phylogenetic analysis using 146 complete genomes of IBV strains downloaded from GenBank was conducted to investigate their relationships with the sequences of our five IBV isolates. The sequences of our five IBV isolates showed close relationships with the Conn vaccine (93.5–93.7%), Cal56b (93.5–93.7%), and IBS037A/2014 (93.9–94.1%) strains. The nucleotide-based MAFFT alignment of our five IBV isolates with the Conn and 4/91 vaccine strains provided six fragments that showed alternating similarity with the two reference strains (Figure 3). One fragment, which started at the 3′ end of the replicase gene and ended at the 3′ end of M gene, was different from both the Conn and 4/91 vaccine strains. Similarity searches using different parts in this fragment showed variable identities with other IBV reference and vaccine strains, where gene 3 revealed a higher sequence similarity of 96.63–96.93% with Mass41 2006 (FJ904713). The highest similarity with the M gene was with ck/CH/LSD/03I (KX236001), which was 96.76–97.20%. The examination of the S gene sequence revealed overall low similarities with all reference sequences, whereas the highest similarity of 87.40–87.82% was observed with Cal56b (GU393331).

**Figure 4:**
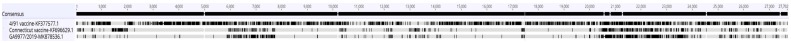
Alignment of the complete genome sequences of isolate GA9977/2019, the Connecticut vaccine, and 4/91 vaccine strains, which was performed using MAFFT. Nucleotide sequence disagreements at indicated positions are shown in black, while nucleotide sequence agreements are shown in gray.

Isolate GA9977/2019 showed 94.7% nucleotide sequence identity with our five IBV isolates, and a 95.5% identity with the Conn vaccine strain. Multiple sequence alignment data, constructed with MAFFT, showed high similarity between isolate GA9977/2019 and the Conn vaccine strain, except for S gene region (Figure 4).

### 3.3. Recombination Analysis

**Figure 5:**
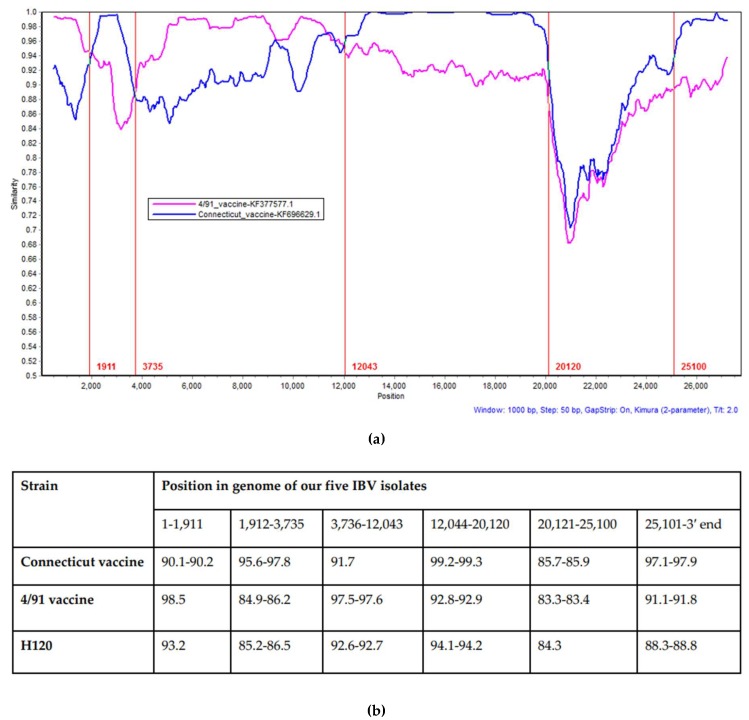

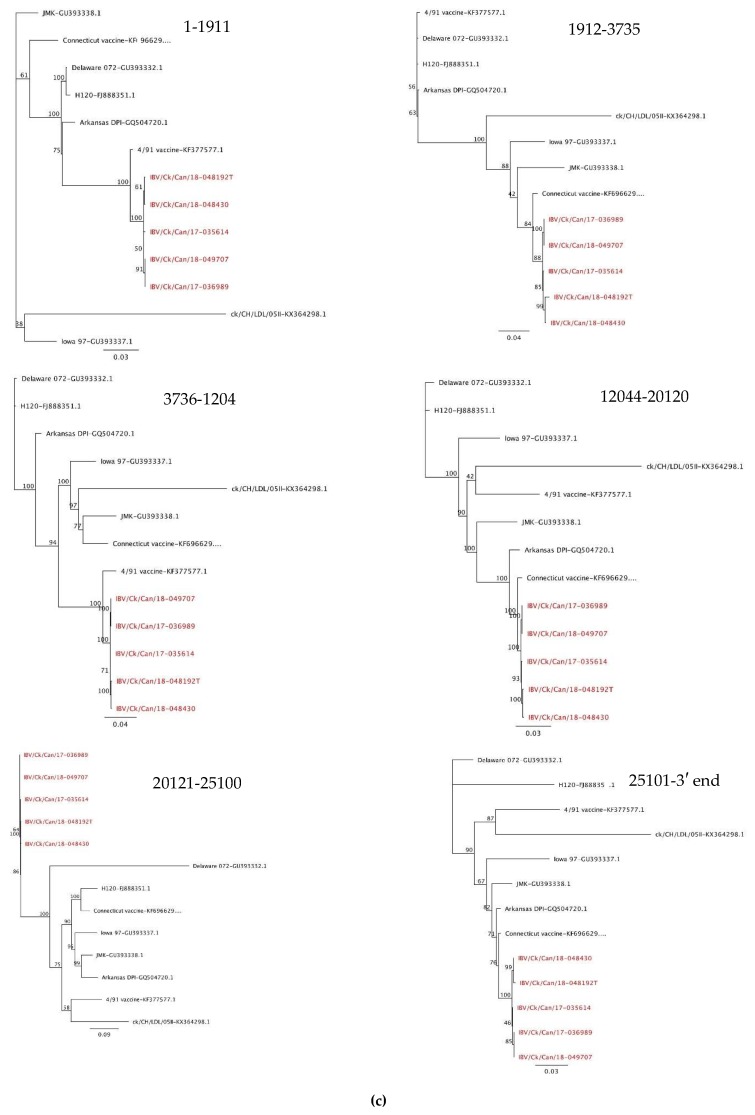
(**a**) Recombination analysis by SimPlot of the complete genome sequences of IBV/Ck/Can/18-049707, the Connecticut vaccine, and the 4/91 vaccine strains. Breakpoints are indicated by the vertical red lines. The SimPlot was created using a window size of 1000 nt and a step size of 50 nt. Isolate IBV/Ck/Can/18-049707 was used as the query sequence. (**b**) Percentages of nucleotide sequence identity among our five IBV isolates, the Connecticut vaccine, the 4/91 vaccine, and H120 (as a representative of the Massachusetts type, the second major live vaccine available in Canada) according to the genome fragments indicated by SimPlot. Genome positions were determined according to isolate IBV/Ck/Can/18-049707 in the alignment. (**c**) Phylogenetic analysis of genome positions 1–1911, 1912–3735, 3736–12043, 12044–20120, 20121–25100, and 25,101–3′ end of the complete genome among our five IBV isolates, Connecticut vaccine, 4/91 vaccine, Arkansas DPI (GQ504720), H120 (FJ888351), JMK (GU393338), Iowa 97 (GU393337), Delaware 072 (Gu393332) and ck/CH/LDL/05II (kx364298). The trees were constructed using the maximum likelihood method in PhyML. Our five IBV isolates are marked in red color.

The Conn vaccine and 4/91 vaccine sequences were used as putative parents, and each sequence of our five IBV isolates was queried in SimPlot. The result of SimPlot confirmed the result of the MAFFT alignment, where six fragments were changing identity with the Conn vaccine and 4/91 vaccine strains, except for the genomic region between 20,120–25,100 nucleotides, which showed low identity with either of these strains. The SimPlot results for our five IBV isolates were identical, and only representative analysis of IBV/Ck/Can/18-049707 is shown (Figure 5a).

Five crossover points were found: The first recombination breakpoint (at approximately 1911 nucleotide position) was located in the nsp2 gene, the second in the nsp3 gene (at approximately the 3735 nucleotide position), the third in the nsp12 gene (at approximately the 12,043 nucleotide position), the fourth at the 3′ end of 1ab gene (at approximately the 20,120 nucleotide position), and the last breakpoint was located at the 3′ end of the M gene (at approximately the 25,100 nucleotide position). In addition, data from the nucleotide similarities and phylogenetic trees constructed using the corresponding gene fragments also confirmed the results (Figure 5b,c).

## 4. Discussion

Infectious bronchitis is one of the most economically important viral diseases that affects poultry worldwide, including in Canada. Like most RNA viruses, the causative agent, the IBV, is genetically diverse, due to a high mutation rate and several recombination events [31]. The aim of the present study was to genetically characterize an IBV variant that has been circulating in Canadian poultry flocks in recent years [15]. Our findings indicated that the S1 genes of our five IBV isolates are similar to the DMV/1639 strain which was originally isolated from an IB outbreak in the Delmarva peninsula in the USA [32]. Further analysis included the elucidation of the complete genome sequences of our five IBV isolates, which demonstrated genetic evidence of recombination with the Conn vaccine, 4/91 vaccine and an unknown IBV strain.

The level of egg passage of the five IBVs used in this study was low (≤4 passages), which decreases the likelihood of genetic changes that could have been introduced as a result of propagation in embryonated eggs. In a study that examined a highly variable region within the IBV genome, namely, the S1 gene, even 10 passages in embryonated eggs resulted in no changes in five viruses. Additionally, two viruses had only 2 nucleotide changes and one virus had only 1 nucleotide change [33]. The genome organization of our five IBV isolates (5′-1a-1ab-S-3a-3b-E-M-4b-4c-5a-5b-N-6b-3′) is consistent with many of the recently reported IBV genomes in different locations around the world [34,35,36,37]. We reported a 13-nucleotide deletion in the reading frame of the 6b gene in isolate IBV/Ck/Can/18-048192T. The examination of the raw sequencing data verified the deletions. Natural IBV isolates lacking accessory proteins (3a, 3b, 5a or 5b), or those expressing a truncated 3a protein, have been reported, and the absence of such accessory proteins did not abolish the replication of these viruses [38,39]. Although the roles of these accessory genes are not fully studied, recombinant IBVs with 3 and/or 5 deleted genes exhibited reduced virulence and induced protective immune responses [40]. Similarly, the ORF 4a protein of Middle East respiratory syndrome (MERS) coronavirus affected interferon therapy response [41] and the overexpression of ORF 6 of severe acute respiratory syndrome (SARS) coronavirus induced cell apoptosis [42]. It is possible that these proteins may have analogous roles in IBVs, although further studies are necessary to confirm this possibility.

The S1 gene is the most variable region within the genome and encodes virus-neutralizing and serotype-specific epitopes. Therefore, it is used often for phylogenetic analysis [43]. Classification of IBV into lineages or genotypes relying on the full S1 gene has been proposed [7], and by using this method, our five IBV isolates were clustered within the GI-17 genotype, where they were highly related to MDL_DMV1639. On the other hand, the Mass and Conn types that are used in live IBV vaccines in Canada showed low similarities with our five IBV isolates, which may explain the increase in the isolation rate of DMV/1639-like isolates from poultry farms in Canada [15]. However, vaccine efficacy studies against DMV/1639-like isolates circulating in Canada are required. The occurrence of IBV strains in Canada that are closely related to IBV strains in USA may be explained by the potential role of wild birds in virus transmission across the border [44]. A substantial portion of diagnostic samples (20.5%) genotyped by the Animal Health Laboratory (University of Guelph, ON, Canada) in the early 2000s were US variant-like viruses [18], which also indicates the possibility IBV transmission between the two neighboring countries. It is also possible that the current IBV isolates in Canada originated from a native parental IBV strain, and studies should be directed towards the confirmation of the origin of the current IBV strains. It was suggested that the IBV strain DMV/1639 evolved from a strain of PA/171/99 which was detected in early 1999 in Pennsylvania, where the two isolates shared a 95.6% S1 nucleotide sequence identity [32,45].

In general, recombination events that contribute to the genetic diversity of coronaviruses is believed to result from a template-switching copy-choice mechanism during RNA replication [46]. The Conn vaccine, Cal56b, and IBS037A/2017 strains showed high sequence identity with our five IBV isolates. However, strains Cal56b and IBS037A/2017 were isolated in the USA and Malaysia, respectively, and they have never been isolated in Canada [47,48]. Since the complete genome sequences of our five IBV isolates were 99.4–100% identical, the SimPlot analysis results were identical. Analysis of the recombination events in our five IBV isolates showed that genetic fragments from the Conn vaccine strain could be detected in our five IBV isolates. Live attenuated Conn vaccines are used in the poultry industry, since this is one of the two live attenuated vaccines available in Canada. Our recombination analysis also indicated that the 4/91 vaccine-like virus is a potential parent of our five IBV isolates. Unlike live attenuated Conn vaccines, modified live vaccines based on the 4/91 strain are not licensed in Canada. The incursion of the IBV 4/91 strain in Canadian poultry started in late 2011 [18]. Unfortunately, there were no recent complete genome sequences available to include in our analysis in this instance. It is noteworthy that seven S1 sequences of 4/91 viruses identified in 2011 were 99.2–100% identical amongst themselves and 99.2–99.6% identical to the IBV 4/91 vaccine strain [18]. It is possible that a large segment of such 4/91 viruses contributed in the replicase gene and 5′ UTR of our five IBV isolates. Four out of five recombination events demonstrated in this study were in the first two thirds of the genome. Consistently, regions with the highest incidence of recombination were located in the replicase transcriptase complex [34,49,50,51]. We developed a hypothesis to explain the scenario of the origin of the fragment that involved the S gene, which states that a recombination event replaced this fragment in a Conn vaccine-like virus with a fragment from an as-yet-unidentified parental virus, which has led to the emergence of these variants which include the PA/171/99 and DMV/1639 strains in the USA. Subsequently, when these isolates started to circulate in Canadian poultry, recombination events that involved 4/91 vaccine-like viruses may have happened. The results obtained from the multiple sequence alignments of our five IBV isolates and isolate GA9977/2019 with the Conn vaccine and 4/91 vaccine strains support this hypothesis, however, it cannot be confirmed, since there were no complete genome sequences available for both the PA/171/99 and DMV/1639 strains immediately after they had been isolated.

Since co-infection with two different viruses in a given flock is a prerequisite for recombination between IBV strains [2], it is critical for poultry flocks to apply effective biosecurity measures that limit the introduction of co-circulating viruses. Furthermore, live attenuated IBV vaccine strains may be involved in such co-infections, as shown in this study, therefore, vaccine efficacy studies against IBV variants with potential recommendation for new vaccines are continuously required. It is also important to note that we could not preclude the remote possibility of co-infection in our samples.

In conclusion, although it is practical for most research studies to genotype the isolated IBV strains using the S1 gene, the complete genome sequencing approach gives more information about the evolutionary origin of such strains. This study is the first one that has characterized the complete genome of the DMV/1639-like isolates that have been circulating in Canadian poultry in recent years. Our findings have implications for the control of IB in Canadian poultry flocks and add to the existing knowledge on the molecular epidemiology of IBV infection.

## Figures and Tables

**Table 1:** History of the five infectious bronchitis virus (IBV) isolates used in this study.

IBV Isolate	Type of Sample	Commodity	Age	Size of the Flock	Main Complaint
IBV/Ck/Can/17-035614	Cecal tonsils	Layers	6 weeks	100,000	Uneven flock
IBV/Ck/Can/17-036989	Kidneys	Layers	22 weeks	NA	Drop in egg production
IBV/Ck/Can/18-048192T	Trachea	Broilers	NA	NA	NA
IBV/Ck/Can/18-048430	Tracheal swab	Broilers	34 days	9408	Routine sampling
IBV/Ck/Can/18-049707	Cecal tonsils	Broiler breeders	41 weeks	20,358	Increased mortality

NA = Not available.

**Table 2:** Percent S1 amino acid and nucleotide sequence identity values for the five IBV isolates used in this study and other strains that are circulating in Canada.

	Nucleotide Identity (%)														
		18-048192T	18-049707	18-048430	17-036989	17-035614	DMV/1639	PA/Wolg/98	Qu-mv	Conn	Mass	4/91	CAL1737	CU_82792	DE/072
**Amino Acid Identity (%)**	**18-048192T**		99.5	99.8	99.4	99.7	96.5	90.1	78.8	77.2	77.2	76.0	76.5	56.4	55.8
	**18-049707**	99.3		99.6	99.9	99.7	96.5	90.1	78.6	77.2	77.2	75.8	76.2	56.3	55.6
	**18-048430**	99.6	99.3		99.5	99.8	96.4	89.9	78.7	77.2	77.2	75.3	76.4	56.4	55.8
	**17-036989**	99.1	99.8	99.1		99.6	96.4	90.0	78.5	77.2	77.2	75.7	75.5	55.3	55.6
	**17-035614**	99.4	99.4	99.6	99.3		96.7	89.9	78.6	77.3	77.3	75.8	76.1	56.3	55.6
	**DMV/1639**	94.8	95.2	95.0	95.0	95.3		91.1	79.7	78.4	78.4	76.2	75.1	56.0	55.3
	**PA/Wolg/98**	87.5	87.3	87.1	87.1	87.1	87.5		79.6	78.6	78.6	76.3	76.1	56.5	56.0
	**Qu-mv**	76.3	76.1	76.1	75.9	75.9	75.9	76.1		78.6	79.3	77.0	75.9	57.2	56.8
	**Conn 46**	75.5	76.9	75.6	75.5	75.6	75.3	75.7	74.3		95.1	77.6	80.3	58.7	57.9
	**M41**	73.0	73.3	73.2	73.2	73.3	73.4	73.9	74.2	90.3		78.3	80.6	58.6	57.9
	**4/91**	75.5	75.5	75.3	75.3	75.3	74.7	74.4	75.1	73.9	73.8		77.2	56.2	55.8
	**CAL1737**	72.8	72.8	72.7	72.7	72.8	71.2	71.6	72.3	77.6	78.3	74.4		57.6	57.2
	**CU_82792**	45.2	45.5	45.4	45.5	45.4	44.6	46.1	48..0	48.4	49.3	45.3	47.1		96.3
	**DE/072**	45.7	45.9	45.9	45.9	45.9	45.0	46.6	47.8	48.8	49.5	45.5	48.0	93.6	

Our five IBV isolates are denoted in boldface.

**Table 3:** Genome features of the five IBV isolates used in this study.

	IBV/Ck/Can/17-035614	IBV/Ck/Can/17-036989	IBV/Ck/Can/18-048192T	IBV/Ck/Can/18-048430	IBV/Ck/Can/18-049707
**5′UTR**	1–530 (530 nt)	1–528 (528 nt)	1–528 (528 nt)	1–528 (528 nt)	1–528 (528 nt)
**1a**	531–12,419 (11889 nt^a^-3962 aa^b^)	529–12,417 (11889 nt-3962 aa)	529–12,360 (11832 nt-3943 aa)	529–12,393 (11865 nt-3954 aa)	529–12,417 (11889 nt-3962 aa)
**1ab**	531–20,452 (19922 nt-6639 aa)	529–20,450 (19922 nt-6639 aa)	529–20,393 (19865 nt-6620 aa)	529–20,426 (19898 nt-6631 aa)	529–20,450 (19922 nt-6639 aa)
**S**	20,403–23,903 (3501 nt-1166 aa)	20,401–23,901 (3501 nt-1166 aa)	20,344–23,844 (3501 nt-1166 aa)	20,377–23,877 (3501 nt-1166 aa)	20,401–23,901 (3501 nt-1166 aa)
**3a**	23,903–24,076 (174 nt-57 aa)	23,901–24,074 (174 nt-57 aa)	23,844–24,017 (174 nt-57 aa)	23,877–24,050 (174 nt-57 aa)	23,901–24,074 (174 nt-57 aa)
**3b**	24,076–24,270 (195 nt-64 aa)	24,074–24,268 (195 nt-64 aa)	24,017–24,211 (195 nt-64 aa)	24,050–24,244 (195 nt-64 aa)	24,074–24,268 (195 nt-64 aa)
**E**	24,251–24,580 (330 nt-109 aa)	24,249–24,578 (330 nt-109 aa)	24,192–24,521 (330 nt-109 aa)	24,225–24,554 (330 nt-109 aa)	24,249–24,578 (330 nt-109 aa)
**M**	24,552–25,229 (678 nt-225 aa)	24,550–25,227 (678 nt-225 aa)	24,493–25,170 (678 nt-225 aa)	24,526–25,203 (678 nt-225 aa)	24,550–25,227 (678 nt-225 aa)
**4b**	25,230–25,514 (285 nt-94 aa)	25,228–25,512 (285 nt-94 aa)	25,171–25,455 (285 nt-94 aa)	25,204–25,488 (285 nt-94 aa)	25,228–25,512 (285 nt-94 aa)
**4c**	25,435–25,605 (171 nt-56 aa)	25,433–25,603 (171 nt-56 aa)	25,376–25,546 (171 nt-56 aa)	25,409–25,579 (171 nt-56 aa)	25,433–25,603 (171 nt-56 aa)
**5a**	25,589–25,786 (198 nt-65 aa)	25,587–25,784 (198 nt-65 aa)	25,530–25,727 (198 nt-65 aa)	25,563–25,760 (198 nt-65 aa)	25,587–25,784 (198 nt-65 aa)
**5b**	25,783–26,031 (249 nt-82 aa)	25,781–26,029 (249 nt-82 aa)	25,724–25,972 (249 nt-82 aa)	25,757–26,005 (249 nt-82 aa)	25,781–26,029 (249 nt-82 aa)
**N**	25,974–27,203 (1230 nt-409 aa)	25,972–27,201 (1230 nt-409 aa)	25,915–27,144 (1230 nt-409 aa)	25,948–27,177 (1230 nt-409 aa)	25,972–27,201 (1230 nt-409 aa)
**6b**	27,212–27,436 (225 nt-74 aa)	27,210–27,434 (225 nt-74 aa)	27,153–27296 (144 nt-47 aa)	27,186–27,410 (225 nt-74 aa)	27210–27,434 (225 nt-74 aa)
**3′UTR**	27,437–27,710 (274 nt)	27,435–27,708 (274 nt)	27,297–27,638 (342 nt)	27,411–27,684 (274 nt)	27,435–27,708 (274 nt)

^a^: Nucleotide length; ^b^: Amino acid length.

## Supplementary Materials

The following are available online at https://www.mdpi.com/1999-4915/11/11/1054/s1, Table S1: List of reference IBV sequences used in the S1 sequence analysis; Table S2: The positions for nonstructural proteins in open reading frames 1a and 1ab.
