# Supplementary material for: Delmarva (DMV/1639) Infectious Bronchitis Virus (IBV) Variants Isolated in Eastern Canada Show Evidence of Recombination
Source: Viruses. 2019 Nov 13;11(11):1054. doi: 10.3390/v11111054 (PMC6893544; doi:10.3390/v11111054)
Supplement: Supplementary file 1 [file viruses-11-01054-s001.pdf]

Supplementary Table 1: List of reference IBV sequences used in the S1 sequence analysis.

Reference sequence	GenBank Accession #	Country of origin
**N1/08**	JN176213	Australia
**V18/91**	U29521	Australia
**N4/02**	DQ059618	Australia
**N5/03**	DQ059619	Australia
**N1/62**	U29522	Australia
**V2-02**	DQ490215	Australia
**B1648**	X87238	Belgium
**IBV/Brasil/351/1984**	GU393339	Brazil
**UFMG/1141**	JX182783	Brazil
**Qu_mv**	AF349621	Canada
**Qu16**	AF349620	Canada
**48SD-96VI**	KC577388	China
**QXIBV**	AF193423	China
**CK/CH/LLN/111169**	KF411040	China
**Connecticut vaccine**	KF696629	China
**QS**	JQ250818	China
**SDW**	DQ070840	China
**SDIB781/2012**	KF007209	China
**TC07-2**	GQ265948	China
**SAIBK**	DQ288927	China
**CK/CH/JX/JA09-1**	HQ018890	China
**ck/CH/LHLJ/110664**	JQ739299	China
**GX2-98**	AY251816	China
**ck/CH/LSD/110857**	JQ739375	China
**4/91 vaccine**	KF377577	China
**ck/CH/LSD/110712**	JQ739363	China
**CK/CH/LDL/97I**	EF030995	China
**Q1**	AF286302	China
**Eg/1265B/2012**	KC533682	Egypt
**FR-85131-85**	AJ618985	France
**It/497/02**	DQ901377	Italy
**IZO 28/86**	KJ941019	Italy
**IBV422**	KF809791	India
**V25**	KF757451	India
**Variant 1**	AF093795	Israel
**IS/1201**	DQ400359	Israel
**Variant 2**	AF093796	Israel
**JP8443**	AY296745	Japan
**K620/02**	FJ807944	Korea
**SNU8067**	JQ977697	Korea
**A**	AF151953	New Zealand
**D**	AF151956	New Zealand
**K43**	AF151958	New Zealand

**H120**	FJ888351	The Netherlands
**D274**	X15832	The Netherlands
**V1397**	M21968	The Netherlands
**D1466**	M21971	The Netherlands
**BL-56**	AF352831	Mexico
**Moroccan-G/83**	EU914938	Morocco
**NGA/N544/2006**	FN182269	Nigeria
**NGA/324/2006**	FN182277	Nigeria
**NGA/295/2006**	FN182276	Nigeria
**NER/28/2007**	FN182272	Niger
**RF/01/02**	AJ441314	Russia
**Spain/00/336**	DQ386098	Spain
**Spain/98/313**	DQ064808	Spain
**TP/64**	AY606320	Taiwan
**UK/L-633/04**	DQ901376	United Kingdom
**UK/7/91**	Z83975	United Kingdom
**6/82**	X04723	United Kingdom
**Beaudette**	M95169	USA
**M41**	AY561711	USA
**Conn46 1996**	FJ904716	USA
**Iowa97**	GU393337	USA
**Gray**	L14069	USA
**JMK**	L14070	USA
**PA/5344/98**	AY789947	USA
**Holte**	L18988	USA
**L905**	JQ964070	USA
**SE17**	M99484	USA
**ARK99**	M99482	USA
**ArkDPI**	AF006624	USA
**Arkansas Vaccine**	GQ504721	USA
**CAL99**	DQ912831	USA
**PA/Wolgemuth/98**	AF305595	USA
**AL/6609/98**	AF510656	USA
**MDL_DMV1639**	KX529720	USA
**CA/1737/04**	EU925393	USA
**DMV/5642/06**	EU694402	USA
**GA/13485/2013**	KP085597	USA
**GA/13384/2013**	KM660635	USA
**GA08**	GU301925	USA
**DE/072/92**	U77298	USA
**GA/13055/00**	AF338719	USA
**CU82792**	AF317214	USA
**PA/1220/98**	AY789942	USA

Supplementary Table 2: The positions[a] for nonstructural proteins in open reading frames 1a and 1ab.

	IBV/Ck/Can/17-035614	IBV/Ck/Can/17-036989	IBV/Ck/Can/18-048192T	IBV/Ck/Can/18-048430	IBV/Ck/Can/18-049707
Nsp2	1M-673G	1M-673G	1M-673G	1M-673G	1M-673G
Nsp3	674G-2275G	674G-2275G	674G-2267G	674G-2267G	674G-2275G
Nsp4	2276G-2789Q	2276G-2789Q	2268G-2781Q	2268G-2781Q	2276G-2789Q
Nsp5	2790A-3096Q	2790A-3096Q	2782A-3088Q	2782A-3088Q	2790A-3096Q
Nsp6	3097S-3390Q	3097S-3390Q	3089S-3382Q	3089S-3382Q	3097S-3390Q
Nsp7	3391S-3473Q	3391S-3473Q	3383S-3465Q	3383S-3465Q	3391S-3473Q
Nsp8	3474S-3683Q	3474S-3683Q	3466S-3675Q	3466S-3675Q	3474S-3683Q
Nsp9	3684N-3794Q	3684N-3794Q	3676N-3789Q	3676N-3789Q	3684N-3794Q
Nsp10	3795S-3939Q	3795S-3939Q	3790S-3934Q	3790S-3934Q	3795S-3939Q
Nsp11	3940S-3962G	3940S-3962G	3935S-3957G	3935S-3957G	3940S-3962G
Nsp12	3954R-4879Q	3954R-4879Q	3949R-4874Q	3949R-4874Q	3954R-4879Q
Nsp13	4880S-5479Q	4880S-5479Q	4875S-5474Q	4875S-5474Q	4880S-5479Q
Nsp14	5480G-6000-Q	5480G-6000-Q	5475G-5995-Q	5475G-5995-Q	5480G-6000-Q
Nsp15	6001S-6338Q	6001S-6338Q	5996S-6333Q	5996S-6333Q	6001S-6338Q
Nsp16	6339S-6640M	6339S-6640M	6334S-6635M	6334S-6635M	6339S-6640M
Nsp2	1M-673G	1M-673G	1M-673G	1M-673G	1M-673G
Nsp3	674G-2275G	674G-2275G	674G-2267G	674G-2267G	674G-2275G

[a:] Positions were based on 1ab from IBV Beaudette strain (accession number NC_001451), and modified according to 1ab from TCoV (accession number YP_001941164) and presented as the residue position with 1 being the methionine at the beginning of ORF 1a and 1ab followed by the single letter code for the amino acid at that position.
